# Exergy and environmental sustainability assessment of PV integrated greenhouse cucumber production

**DOI:** 10.1038/s41598-026-66949-5

**Published:** 2026-08-15

**Authors:** Müjdat Öztürk, Hasan Yildizhan, Arman Ameen

**Affiliations:** 1 Department of Mechanical Engineering, Faculty of Engineering and Architecture, Kırşehir Ahi Evran University, Kırşehir, Turkey; 2 Department of Energy Systems Engineering, Adana Alparslan Türkeş Science and Technology University, Adana, Turkey; 3 Department of Building Engineering, Energy Systems and Sustainability Science, University of Gävle, 801 76 Gävle, Sweden

**Keywords:** Energy and exergy analysis, Renewable energy, Cucumber, Greenhouse, Sustainability indicators, Energy science and technology, Engineering, Environmental sciences

## Abstract

This study examines the thermodynamic and environmental performance of cucumber produced under greenhouse conditions in Türkiye with a holistic approach. The analysis, conducted in the provinces of Kırklareli and Konya, calculated cumulative energy consumption (CEnC), cumulative exergy consumption (CExC), and cumulative CO_2_ emissions (CCO_2_E), with a functional unit of one ton of product. In addition, two important sustainability indicators, namely Cumulative Degree of Perfection (CDP) and Renewability Indicator (RI), were calculated based on exergy calculations. According to the results, CEnC was 260.16 MJ/ton, CExC was 1459.82 MJ/ton, and CCO_2_E was 31.13 kg/ton in Kırklareli, while these values were determined to be 324.38 MJ/ton, 2521.12 MJ/ton, and 50.91 kg/ton, respectively, in Konya. This difference indicates that irrigation and electricity use increase the energy and exergy intensity in Konya. Exergy based sustainability indicators, CDP and RI values, were calculated as 0.73 and − 0.36 for Kırklareli, and 0.42 and − 1.36 for Konya, respectively. These values reveal the high dependence of current production processes on non-renewable energy sources. When photovoltaic (PV) panels were used to supply electricity, the CDP in Kırklareli increased from 0.73 to 0.77, and the RI improved from − 0.36 to -0.30. In Konya, the CDP value rose to 0.51, and the RI to -0.96. These results suggest that PV integration has the potential to improve renewability indicators, especially in energy intensive regions. The findings indicate that, within the scope of this analysis, the sustainability of greenhouse cucumber production could be enhanced through optimized resource management and PV systems.

## Introduction

Global population growth is increasing food demand, leaving agricultural systems facing a difficult balance between productivity and sustainability. Increasing production volume is no longer enough to meet this growing demand; it must also be achieved through environmentally and economically sustainable methods. Although modern agriculture has made significant progress in boosting production capacity, its heavy reliance on energy and the resulting environmental impacts from fossil fuels make it imperative to develop new, sustainable approaches^1,2^. While serving as a cornerstone of food security, the agricultural sector also causes environmental impacts such as high energy consumption and greenhouse gas emissions^3,4^. This situation increases the need for sustainable farming practices that use resources efficiently, minimize the environmental footprint, and are economically viable. In this context, greenhouse cultivation, which enables higher yields per unit area and extends the production season, plays a critical role in food supply security.

Türkiye is one of the world’s leading countries in both total vegetable production and greenhouse farming^5,6^. However, greenhouses, particularly those in the Mediterranean climate zone, are systems that require intensive energy consumption for activities like heating, cooling, ventilation, and irrigation^7^, which both increases production costs and leads to environmental problems. Greenhouse cucumber cultivation in Türkiye is widespread across different regions, and energy analyses conducted in this area show significant differences. Among the studied greenhouse vegetables, cucumber stands out as a product with a notably high energy consumption^8^. Studies have reported a wide range for the energy input/output ratio in cucumber production, such as 1.16 to 1.94 in the Thrace region^9^, and 0.76^8^ and 0.31^10^ in Antalya. This variability clearly demonstrates the impact of different climatic conditions, greenhouse technologies, and agricultural practices on energy efficiency. Studies reveal that the largest share of energy consumption typically comes from fossil fuel based, non-renewable sources like diesel fuel, electricity, and nitrogen fertilizers^11–13^. This high dependency both increases production costs and causes significant carbon emissions.

To assess the sustainability of agricultural systems, traditional energy input/output analyses are often used. Based on the first law of thermodynamics the principle of energy conservation these analyses provide a quantitative assessment of energy use but disregard the quality of different energy forms and thermodynamic inefficiencies in the processes. To overcome this limitation, exergy analysis, which combines the first and second laws of thermodynamics, offers a more comprehensive approach^14–16^. Exergy represents the maximum useful work potential that a system can produce as it reaches equilibrium with its surroundings, and it allows for the identification and magnitude of real losses (exergy destruction) in the system by considering energy quality. This characteristic makes exergy analysis a superior tool for evaluating the efficiency and environmental performance of agricultural systems.

Taking exergy analysis a step further, Cumulative Exergy Consumption (CExC) calculates the total exergy consumed throughout a product’s entire life cycle, from raw material extraction to the final product^17^. Two important sustainability indicators are calculated based on CExC: Cumulative Perfection Degree (CDP) and the Renewability Indicator (RI). CDP is the ratio of the chemical exergy of the final product to the total exergy of the non-renewable resources consumed during the production process, and it indicates the efficiency of a production technique. RI, on the other hand, is a metric that reveals the extent to which a process relies on renewable resources^18,19^. These indicators provide powerful analytical tools for a deep understanding of the environmental compatibility and sustainability level of agricultural production processes.

Traditionally, analyses of cucumber production processes have predominantly relied on the first law of thermodynamics. This study builds upon existing methodologies to provide a regional assessment of thermodynamic performance for specific greenhouse cucumber production prototypes in Türkiye. By focusing on Kırklareli and Konya as case studies, the analysis offers insights into how varying climatic conditions influence exergy-based sustainability indicators. By integrating sustainability metrics such as CDP and RI, the analysis offers a detailed assessment of resource quality and renewability. Kırklareli and Konya were selected as case studies to represent humid and semi-arid climatic characteristics, respectively, offering insights into how varying environmental conditions influence thermodynamic performance. Furthermore, the study explores the potential impact of integrating photovoltaic (PV) systems into greenhouse operations to assess improvements in operational sustainability. While the findings are rooted in these specific regional prototypes, the integrated evaluation of energy quantity, quality, and emission impact provides a valuable reference for developing location-specific strategies for sustainable greenhouse production.

## Methodology

This section details the methodology used to comparatively analyze the energy, exergy, and environmental performance of greenhouse cucumber production in Türkiye’s important agricultural regions of Kırklareli and Konya, which have different climatic characteristics. The study consists of the following stages: defining the research areas and analyzing their climatic features, data collection, thermodynamic analysis, and calculating sustainability indicators.

### Defining research areas and their climatic features

The study was conducted in the provinces of Kırklareli and Konya, which have distinct characteristics in terms of greenhouse farming in Türkiye. The main goal of selecting these provinces was to compare the effects of different climatic conditions and associated agricultural practices on energy efficiency and sustainability indicators.

Kırklareli: Located in the Thrace region, approximately 41% of Kırklareli’s total area consists of agricultural land. The region’s agricultural structure is primarily based on products such as cereals, sunflower, corn, and forage crops. Production is carried out under dry farming conditions in 83% of the province’s agricultural land^20^. This indicates that agricultural activities are largely dependent on rainfall and adopt a less intensive irrigation model.

Konya: Located in the Central Anatolia region, Konya has a continental climate. It is one of the least rainy regions in Türkiye, and limited water resources are the most significant factor restricting agricultural production. Approximately 45% of Konya’s total area is used for agriculture (with 67.6% being dry farming), which corresponds to about 8.04% of Türkiye’s total agricultural land. The province is a leader in Türkiye for the production of wheat, barley, chickpeas, dry beans, sunflower, and especially industrial crops like sugar beets^21^. Agricultural irrigation in the region is largely carried out by drawing groundwater from deep wells. This leads to intense electricity consumption for water pumping. Additionally, Konya ranks first in Türkiye in terms of cattle numbers. This large livestock sector also provides a significant source of organic fertilizer for agricultural production.

### Data collection

The data used in this study are based on data obtained from references^9,22^ regarding greenhouse cucumber production in Kırklareli and Konya provinces. The specific amounts of agricultural inputs required for one ton of cucumber production in both Kırklareli and Konya are categorically detailed in Table 1, which serves as the primary data foundation for the subsequent analysis. The collected data includes inputs such as diesel fuel, electricity, chemical fertilizers, manure, chemicals, and irrigation water, as well as the total cucumber production.

In this study, all input parameters and thermodynamic coefficients used were obtained from validated literature sources. The accuracy of the findings is based on the reliability of the peer-reviewed datasets and formulas cited throughout the text.

The analysis was conducted by considering all primary inputs from “tillage to harvest” (including fertilizers, manure, chemicals, diesel, electricity, and irrigation water), using “one ton of cucumber production” as the functional unit. The system boundary excludes post-harvest stages, packaging, and storage.

**Table 1:** Distribution and amounts of inputs in cucumber production under greenhouse conditions (Data compiled from^9,22^).

Inputs		Unit	Input quantities for cucumber production per ton	
			Kırklareli^9^	Konya^22^
Fertilizers	Nitrogen (N)	kg	0.868	0.239
	Phosphorus (P_2_O_5_)	kg	0.269	0.687
	Potassium (K_2_O)	kg	0.612	0.261
Manure		kg	226.492	347.826
Chemicals	Insecticides	kg	0.122	0.004
	Fungicides	kg	0.056	0.022
Diesel		kg	0.269	0.146
Electricity		MJ	21.209	126.125
Water for irrigation		kg	27.179	28.043

In this study, the inputs for the greenhouse cucumber production process in Kırklareli and Konya provinces were calculated using specific cumulative energy consumption (CEnC), CExC and cumulative CO_2_ emissions (CCO_2_E) coefficients obtained from the literature. The analysis includes the unit energy, exergy and emission coefficients presented in Table 2, allowing for a comprehensive evaluation of each input. The agricultural inputs required for 1 ton of production in Table 1 were considered within the scope of the analysis. The energy and exergy coefficients represent the resource consumption that occurs throughout the entire life cycle of the inputs, from the production stage to their use on the farm.

The coefficients used in the study also enable the evaluation of the environmental impacts of the inputs used in greenhouse production. In this context, carbon dioxide emission coefficients are included in the calculations to determine each input’s effect on global warming. Therefore, this analysis, conducted using the data presented in Table 2, allows for a comprehensive assessment of the energy and exergy consumption levels, as well as the carbon footprint, for cucumber production in Kırklareli and Konya. This approach not only determines the efficiency level of the production process but also reveals the current state of greenhouse based agricultural activities in terms of environmental sustainability.

**Table 2:** Unit energy, exergy and carbon dioxide emission coefficients of inputs used in cucumber production under greenhouse conditions.

Inputs	Specific CEnC	Specific CExC	Specific CCO_2_E
Fertilizers			
Nitrogen (N)	78.2 MJ/kg^23^	32.7 MJ/kg^17^	0.09 kg/kg^24^
Phosphorus (P_2_O_5_)	17.5 MJ/kg^23^	7.52 MJ/kg^25^	0.15 kg/kg^24^
Potassium (K_2_O)	13.8 MJ/kg^23^	4.56 MJ/kg^26^	0.51 kg/kg^24^
Chemicals			
Insecticides	198.8 MJ/kg^27^	7.5 MJ/kg^25^	5.1 kg/kg^28^
Fungicides	198.8 MJ/kg^27^	4.6 MJ/kg^26^	3.9 kg/kg^28^
Manure	0.35 MJ/kg^29^	5.33 MJ/kg^30^	0.0462 kg/kg^30^
Diesel	57.5 MJ/kg^27^	53.2 MJ/kg^17^	0.94 kg/kg^28^
Electricity	1 MJ/MJ^17^	4.17 MJ/MJ^17^	0.14 kg/MJ^31^
Water for irrigation	0.00102 MJ/kg^27^	0.00425 MJ/kg^30^	0.000595 kg/kg^30^

**Fig. 1:**
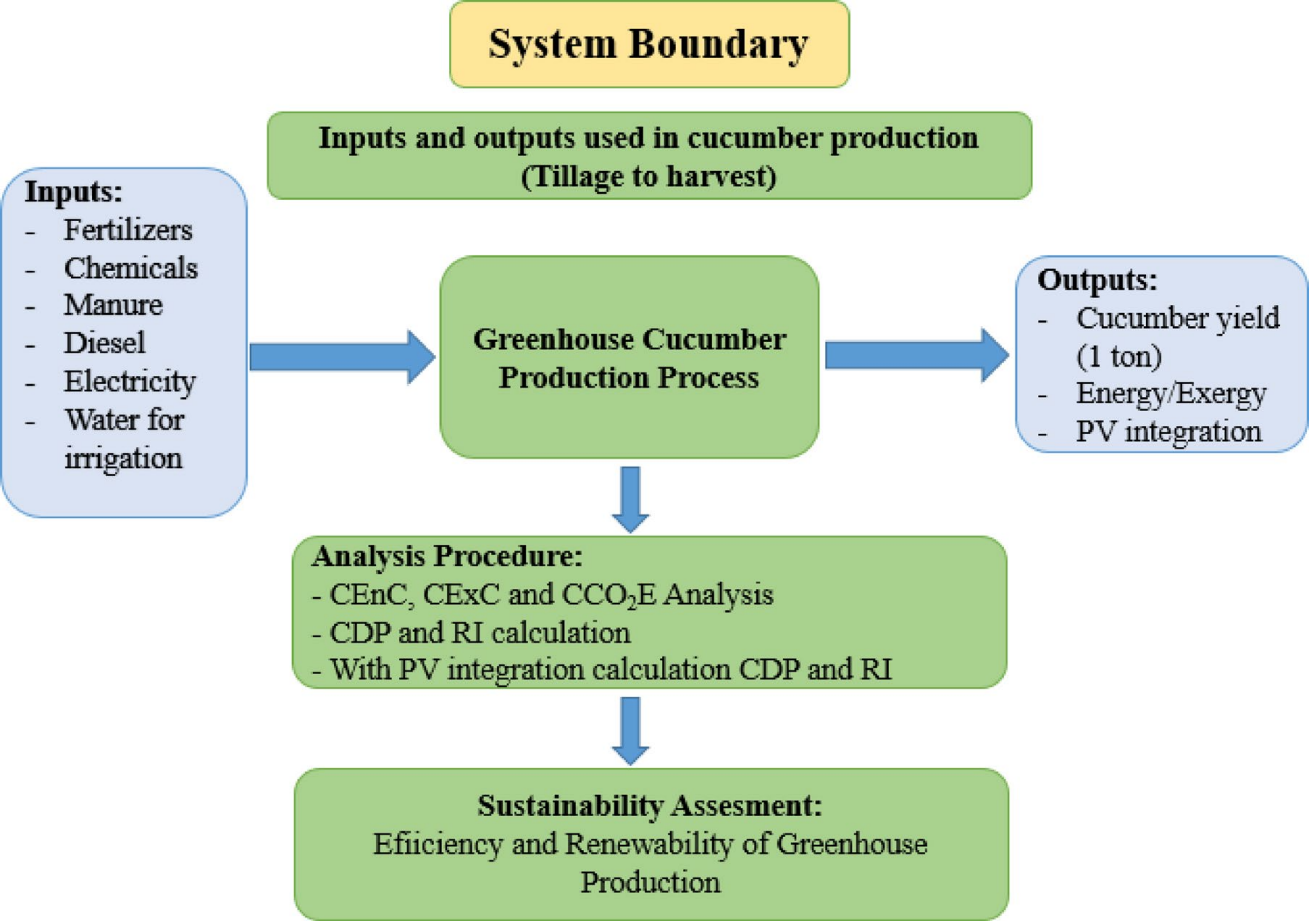
System boundary, research procedure, and sustainability assessment of one ton of cucumber production.

To evaluate the sustainability of cucumber production under greenhouse conditions, the system boundary and research procedure must first be clearly defined. Figure 1 illustrates the general framework of the study, showing the main inputs, the production process, the outputs, and the subsequent analysis stages. Within the defined system boundaries, agricultural inputs such as fertilizers, chemicals, manure, diesel, electricity, and irrigation water are included. These inputs directly affect the energy, exergy, and environmental performance of the greenhouse production process. The analyses were calculated based on 1 ton of cucumber product, and the results were expressed as CEnC, CExC, and CCO_2_E.

Following the production process, the data obtained is subjected to a detailed evaluation, including energy and exergy analyses, as well as the calculation of CO_2_ emissions. Based on the results of these analyses, advanced indicators such as CDP and RI are calculated to quantitatively reveal the system’s efficiency and sustainability performance. An analysis is also presented to increase efficiency and determine the renewability potential of the cucumber production process under a scenario where PV panels are installed in the greenhouses, based on the solar energy potential of the provinces.

### Thermodynamic analysis

The energy efficiency of the agricultural production system was evaluated based on the ratio between energy inputs and outputs. The collected physical input and output data were converted into the energy unit of Megajoules (MJ) using widely accepted energy equivalent coefficients from the literature.

To more deeply analyze inefficiencies and resource quality in production processes, exergy analysis, based on the first and second laws of thermodynamics, was applied. This approach is based on the concept of CExC, which represents the total exergy consumed throughout a product’s life cycle^17^. The analysis is based on fundamental mass, energy, and exergy balance equations^32^.

Mass Balance:

1 $$\:{\Sigma\:}\left(m\right)\mathrm{i}\mathrm{n}\:=\:{\Sigma\:}\left(m\right)\mathrm{o}\mathrm{u}\mathrm{t}$$

Energy Balance (I. Law):

2 $$\sum {(mh)_{{in}} } - \sum {(mh)_{{out}} } = Q - W$$

Exergy Balance (II. Law):

3 $$\sum {(mB)_{{in}} } - \sum {(mB)_{{out}} } + \sum {(1 - \frac{{T_{o} }}{{T_{k} }})} Q_{k} - W = X_{{loss}}$$

In these equations, *m* represents mass, *h* enthalpy, *b* flow exergy, *W* work, *Q* heat, *T* temperature, and *I* represents exergy destruction^33,34^.

4 $$b = b^{{ch}} + {\text{ }}b^{{th~}}$$

In this study, the chemical exergy is represented by *b*^*ch*^, whereas the physical exergy is denoted by *b*^*th*^.

5 $$b^{{th}} = {\text{ }}h{\text{ }}{-}T_{0} s$$

Cumulative Perfection Degree (CDP):

It shows the thermodynamic efficiency of the process by expressing the ratio of the product’s chemical exergy to the total exergy consumed^17^;

6 $$\:CDP=\frac{{\left(\mathrm{m}\mathrm{b}\right)}_{\mathrm{p}\mathrm{r}\mathrm{o}\mathrm{d}\mathrm{u}\mathrm{c}\mathrm{t}}}{\sum\:{\left(m\mathrm{C}\mathrm{E}\mathrm{x}\mathrm{C}\right)}_{\mathrm{r}\mathrm{a}\mathrm{w}\:\mathrm{m}\mathrm{a}\mathrm{t}\mathrm{e}\mathrm{r}\mathrm{i}\mathrm{a}\mathrm{l}\mathrm{s}\:}+\sum\:{\left(m\mathrm{C}\mathrm{E}\mathrm{x}\mathrm{C}\right)}_{\mathrm{f}\mathrm{u}\mathrm{e}\mathrm{l}\mathrm{s}\:}}$$

Here, CExC is a fundamental concept for analyzing the sustainability performance of systems. Expanding on this approach, new indicators such as net exergy consumption (CNEx) and restoration work (W_r_) have been determined^18^.

Net exergy consumption is expressed as the total cumulative exergy loss that occurs throughout a process, making it possible to evaluate a system’s efficiency and environmental impacts more comprehensively.

7 $$\:CNEx=CExC-{Ex}_{p}$$

8 $$\:{W}_{r}={CNEx}_{p}-{CNEx}_{waste}$$

Here, CNEx_p_ represents non-renewable energy consumption, while CNEx_waste_ indicates the amount of energy required for waste disposal. The chemical exergy of the product is represented by Ex_p_^18^.

Renewability Indicator (RI).

Evaluates the ratio of the produced chemical exergy to the work required to restore non-renewable resources and indicates how renewable the process is^18^.

9 $$\:RI=\frac{{W}_{P}-{W}_{r}}{{W}_{p}}$$

RI is an indicator defined based on the ratio of the useful work (W_p_) obtained during the system’s production process to the restoration work. This index reveals the renewability level of the production process. The process descriptions corresponding to different ranges of RI values are more clearly shown in Table 3. This visual summarizes how RI values determine the level of renewability in a process^18^.

**Table 3:** Process classification based on RI value.

RI Value	Process description
RI = 1	Completely renewable process (RI = 1): In this case, no external restoration work (Wr) is performed on the system. The process has the ability to sustain itself.
0 < RI < 1	Partially renewable process (0 < RI < 1): Although the process has renewability features, it is not entirely self-sufficient. A certain amount of restoration work (Wr) is required to sustain the process.
RI = 0	Process where produced work equals restoration work (RI = 0): In this equilibrium state, the work produced during the process is exactly equal to the restoration work supplied externally to the system.
RI < 0	Process where restoration work is greater than produced work (RI < 0): This is a completely non-renewable process.

## Results and discussion

This study provides an in depth examination of the intensive resource utilization and environmental impacts of modern greenhouse farming by employing thermodynamic and exergy based analyses. The focal point of this work is to assess the greenhouse cucumber production process in Türkiye through a holistic approach, by analyzing energy consumption, exergy consumption, and carbon dioxide emissions via key metrics such as CDP and RI. The analyses consider one ton of greenhouse cucumber production as the functional unit. The data obtained provides a holistic evaluation of where greenhouse cucumber production is positioned in the context of energy efficiency and environmental performance, through comparison with an extensive body of literature on other agricultural production processes.

### Cumulative energy consumption (CEnC) analysis

**Fig. 2:**
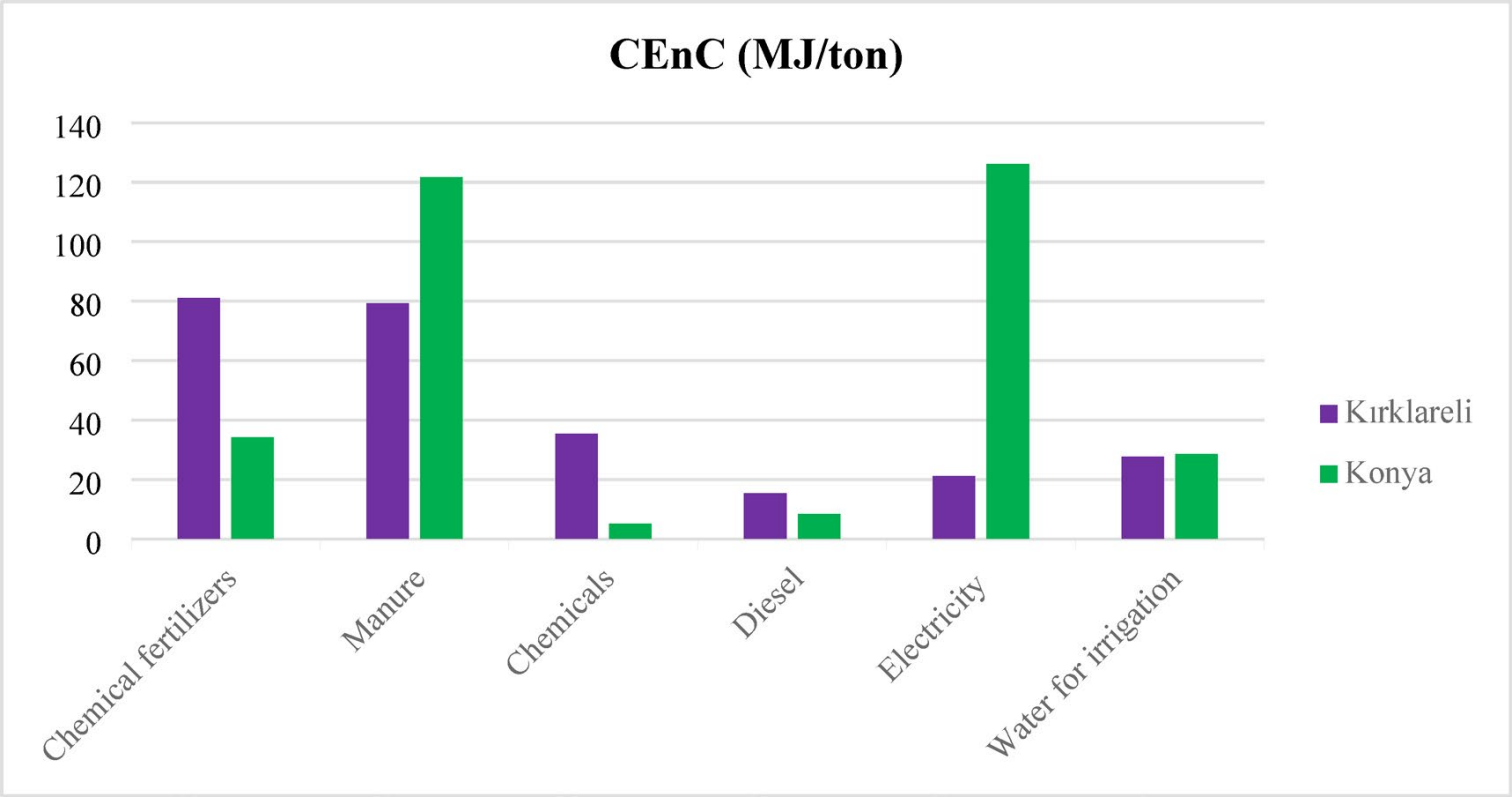
Cumulative energy consumption according to inputs of one ton cucumber production.

This section details the per ton cumulative energy consumption for greenhouse cucumber production in Kırklareli and Konya, two distinct agricultural regions in Türkiye. CEnC analysis provides a critical thermodynamic metric for assessing the energy intensity and sustainability performance of agricultural production processes. As illustrated in the comparative distribution in Fig. 2, the distribution of energy consumption reveals that primary energy drivers shift significantly depending on the regional context.

According to the data obtained, the total energy consumption in Konya is significantly higher compared to Kırklareli, primarily driven by differences in manure, electricity, and irrigation water inputs. As shown in Table 1, the higher manure consumption in Konya suggests a need for more intensive nutrient supplementation. This trend of high fertilizer impact is a common bottleneck in agricultural energy budgets; for instance, in onion production in Iran, nitrogen fertilizers were found to be the largest contributor to CEnC, accounting for up to 44.64% of total energy use^35^. Furthermore, findings by Degerli et al. (2015) and Öztürk et al. (2025) recent assessments of agricultural production confirm that nitrogen fertilizers, due to their energy-intensive production processes, remain a dominant factor in energy consumption across different crop types^36,37^.

One of the most notable differences is observed in electricity and irrigation water consumption. In Konya, 126.13 MJ/ton is spent on electricity and 28.60 MJ/ton on irrigation, while in Kırklareli, these values are 21.21 MJ/ton and 27.72 MJ/ton, respectively. The high electricity demand in Konya is consistent with its semi-arid continental climate and reliance on deep groundwater extraction. This parallels findings in apple cultivation in Niğde, where water scarcity and deep-well pumping resulted in an irrigation energy demand (111.43 MJ/ton) nearly 20 times greater than in more humid regions like Antalya (5.87 MJ/ton)^37^. Hesampour et al. (2022) stated that in regions where diesel or electric pumps are used for water extraction, these activities constitute the primary source of total energy consumption^38^. Similarly, a study on maize production found that irrigated systems have much higher energy consumption than rain fed systems^16^.Regarding the CEnC profile, chemical fertilizers in this study (Konya: 34.32 MJ/ton, Kırklareli: 81.03 MJ/ton) show lower total consumption compared to manure. In a similar thermodynamic assessment of greenhouse cucumber production, Taki and Yıldızhan (2018) reported a significantly higher total CEnC of 4412.39 MJ/ton, with 48.5% attributed to diesel fuel^39^.

While the diesel consumption in the current study (Konya: 15.45 MJ/ton, Kırklareli: 8.40 MJ/ton) is lower, it aligns with the broader observation that fossil fuel based inputs are decisive factors in energy intensity. The integration of renewable energy scenarios, such as the use of liquid hydrogen in onion cultivation^35^ or agricultural photovoltaic systems in apple cultivation^37^, has been shown to significantly reduce such energy consumption; highlighting that this is a critical way to improve the sustainability of these input-intensive agricultural ecosystems.

The data obtained reveal distinct structures of energy consumption in greenhouse cucumber production across the studied regions of Kırklareli and Konya. The higher CEnC values observed in Konya are primarily associated with significant electricity demand and more intensive fertilization practices. These findings suggest that for similar semi-arid climatic contexts, sustainability strategies could prioritize enhancing electricity use efficiency and exploring the integration of renewable energy sources, such as solar-powered pumping, specifically for operational energy substitution. Conversely, the lower CEnC values in Kırklareli reflect regional climatic conditions that necessitate lower irrigation and energy intensity. For both provincial prototypes, the adoption of precision farming techniques and optimized fertilizer management shows significant potential for reducing energy consumption and mitigating environmental impacts within the defined production boundaries.

### Cumulative exergy consumption (CExC) analysis

**Fig. 3:**
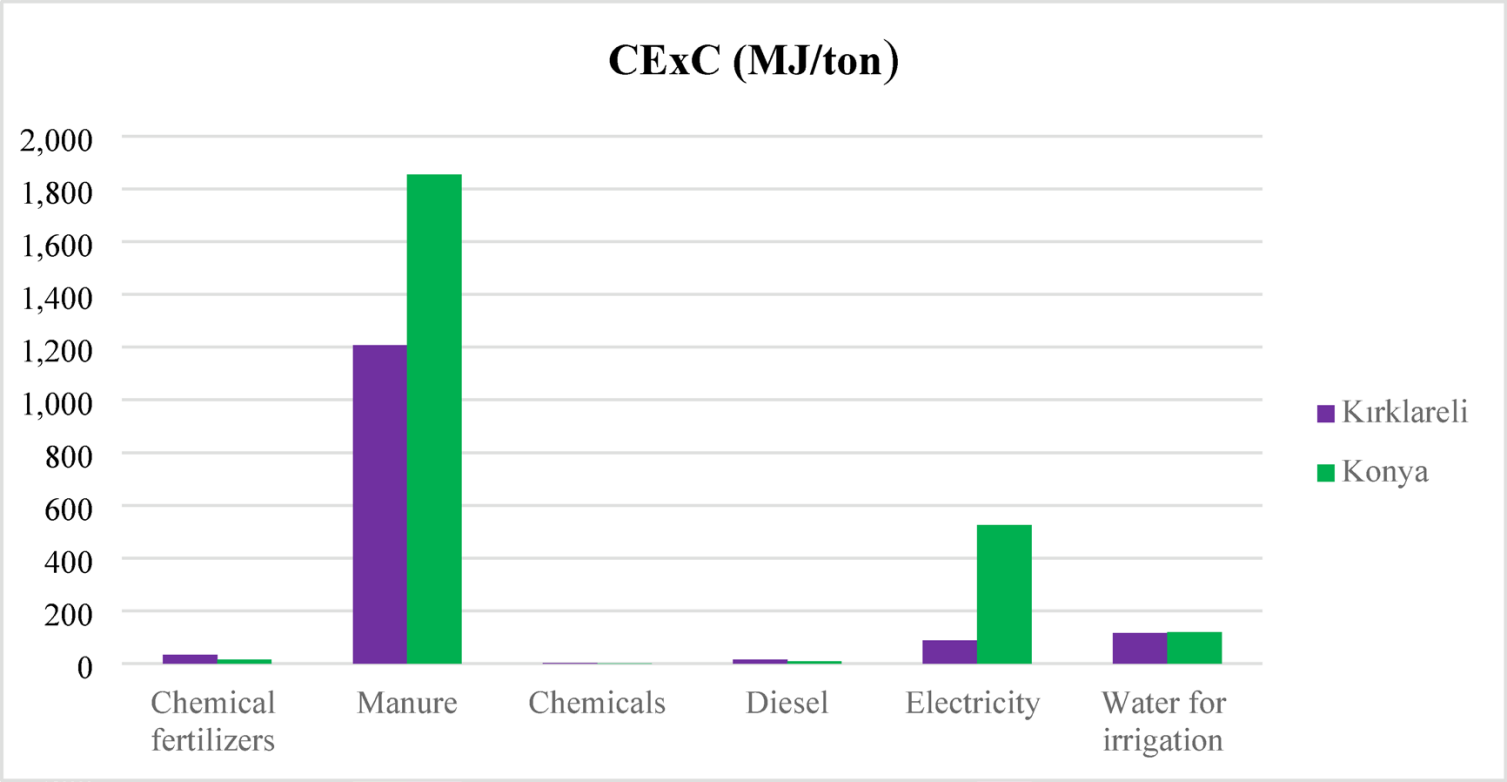
Input based cumulative exergy consumption in the production of one ton of cucumber.

Exergy analysis is an advanced method that evaluates a system’s true thermodynamic efficiency by considering not only the quantity of energy but also its potential to perform useful work. Figure 3 reveals that different inputs show significant differences in exergy consumption between Kırklareli and Konya provinces.

The results indicate that Konya has a significantly higher total CExC value in greenhouse cucumber production compared to Kırklareli. This difference becomes evident particularly in key input items such as manure, electricity, and irrigation water. The largest exergy consumption in both provinces stems from manure. However, this value was calculated at 1853.91 MJ/ton in Konya, while it was 1207.20 MJ/ton in Kırklareli.

The difference in manure application rates stems from regional soil and climate variations. Semi-arid Konya requires high manure input to compensate for low organic matter and increase water retention capacity. In contrast, Kırklareli’s humid climate and superior soil profile necessitate more moderate organic fertilization. These agricultural adaptations significantly alter cumulative energy and exergy inputs due to the high exergy density of the manure.

The dominant role of manure in exergy consumption is consistent with analyses by Özilgen and Sorgüven (2011), who emphasize the high potential of the chemical structure of organic inputs^32^. This result demonstrates that biological inputs like fertilizer have a high thermodynamic potential that can be overlooked in a simple energy balance analysis but is revealed through exergy analysis.

Electricity consumption also shows a significant difference between the two provinces. The exergy consumption for electricity, at 525.94 MJ/ton in Konya, is approximately six times higher than the 88.44 MJ/ton value in Kırklareli. Similarly, the exergy spent on irrigation water is also slightly higher in Konya (119.18 MJ/ton) than in Kırklareli (115.51 MJ/ton). This situation is due to the vital importance of irrigation in Konya’s arid climate and the high energy cost of extracting water from deep wells.

A similar regional disparity was observed in apple production, where Niğde exhibited significantly higher exergy consumption (2975 MJ/ton) compared to Antalya (1183 MJ/ton), primarily due to diesel-powered irrigation and deep-well pumping in more arid zones^37^. Furthermore, a study on date production by Hesampour et al. (2022) stated that in regions where diesel pumps are used for water extraction and irrigation, these activities constitute the largest source of the total CExC^38^. In contrast, in more modern irrigation infrastructures, such as those analyzed for onion production, the transition to renewable energy sources like liquid hydrogen can reduce exergy consumption significantly^35^.

The share of chemical fertilizers in total exergy consumption is considerably lower than that of manure. In their study on vegetable oil production from sunflower and soybeans, Özilgen and Sorgüven (2011) stated that the highest CExC resulted from fertilizer use, but this was due to excessive and inefficient application^32^. The relatively low chemical fertilizer exergy consumption in greenhouse cucumber production indicates that these inputs are used more efficiently.

According to the findings from the analyses, the exergy profiles in greenhouse cucumber production in Kırklareli and Konya differ due to regional climate and farming practices. The higher CExC observed in Konya is primarily associated with energy-intensive activities, particularly electricity consumption for deep-well pumping and manure utilization. These results indicate that exergy analysis, by accounting for the quality and efficiency of resources, offers a more detailed thermodynamic perspective than traditional energy analyses for assessing the sustainability of regional greenhouse cucumber production. Consequently, sustainability efforts in semi-arid regions similar to Konya could prioritize the optimization of irrigation systems and the exploration of renewable energy integration, specifically for operational energy substitution within the defined production boundaries.

### Cumulative carbon dioxide emissions (CCO_2_E) analysis

**Fig. 4:**
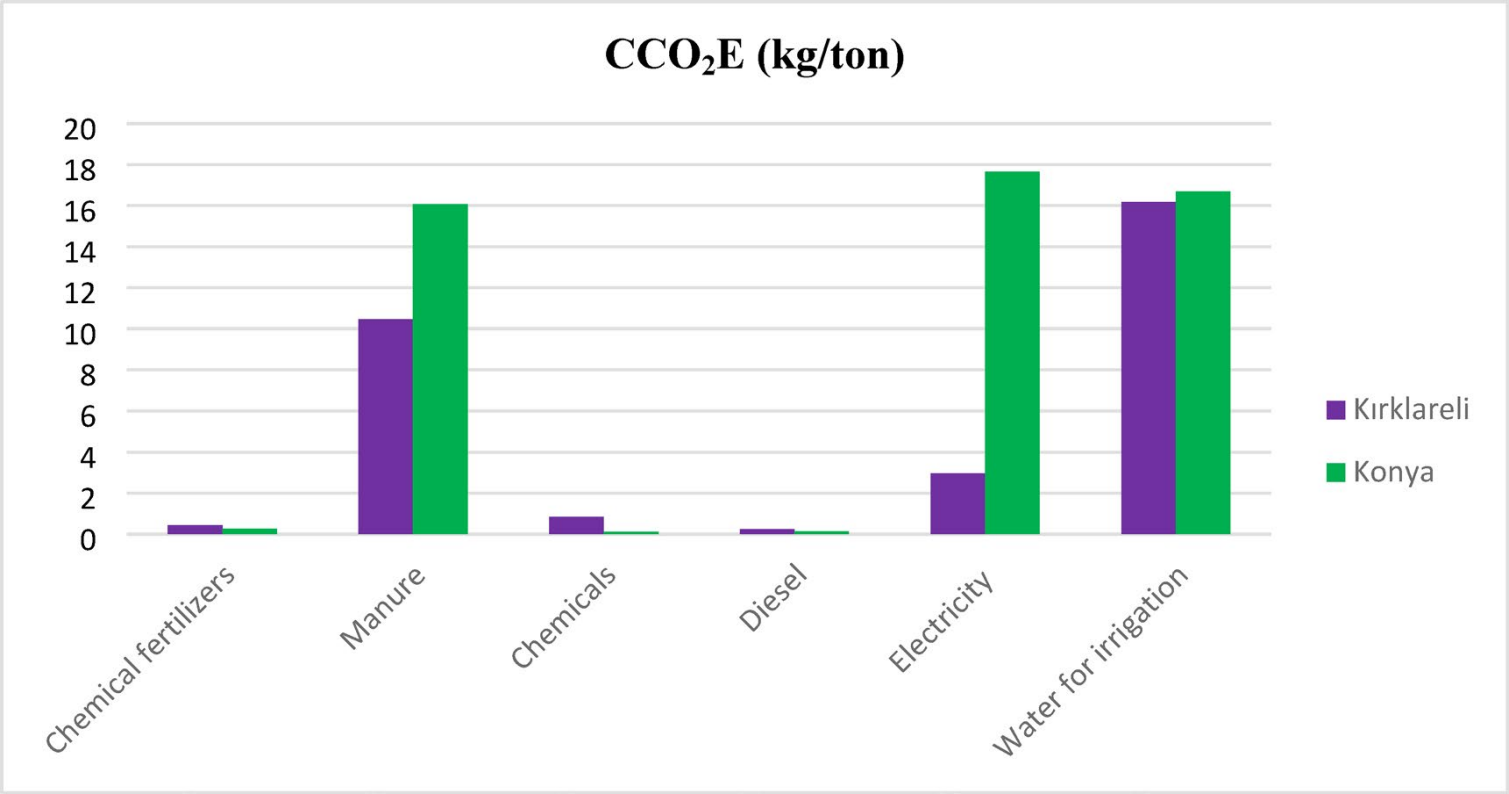
Cumulative CO_2_ emissions associated with 1 ton of greenhouse cucumber production.

Figure 4 shows the impact of inputs used for producing one ton of greenhouse cucumber in Kırklareli and Konya, Türkiye’s different climate regions, on carbon dioxide emissions. CCO_2_E is a key metric for determining a product’s environmental sustainability and carbon footprint.

While the inputs that contribute the most to total emissions differ in each province, some common trends are observed. Emission values in Konya’s production process are notably higher than in Kırklareli for many inputs. For example, manure use in Konya generates 16.07 kg CO_2_/ton of emissions, while this value is at 10.46 kg CO_2_/ton in Kırklareli. Similarly, electricity consumption is significantly higher in Konya at 17.66 kg CO_2_/ton compared to 2.97 kg CO_2_/ton in Kırklareli. This situation indicates that electricity intensive activities in Konya substantially increase the environmental footprint of greenhouse cucumber production. This finding is consistent with a study on maize production in Mexico, which revealed that irrigated systems have a larger environmental footprint due to high input use^16^.

According to the analysis, inputs such as irrigation water, manure, and electricity contribute the most to total emissions in both provinces. In Konya, electricity (17.66 kg CO_2_/ton) and irrigation water (16.69 kg CO_2_/ton) are the highest emission sources, whereas in Kırklareli, the highest emissions come from farm manure (10.46 kg CO_2_/ton) and irrigation water (16.17 kg CO_2_/ton). These data suggest that within the studied regional contexts, strategies to enhance environmental sustainability in greenhouse cucumber production could effectively prioritize the efficiency of manure, water, and electricity use. Within the defined tillage-to-harvest assessment boundaries, optimizing these specific inputs appears to be a critical factor for mitigating the carbon footprint of the production phase in both semi-arid and humid climatic prototypes.

This regional emission profile is consistent with findings in apple production in Niğde and Isparta, where irrigation water and diesel-powered pumping were identified as the primary drivers of carbon intensity, reaching values up to 65.49 kg CO_2_/ton^37^. Furthermore, a study by Taki and Yildizhan (2018) reported that the total CCO_2_E value for greenhouse cucumber production in Iran was approximately 175 kg CO_2_/ton, with 64% of it originating from natural gas used for heating^37^. While this is higher than the emission values calculated in this study, the decisive role of key greenhouse farming inputs on emissions emerges as a common finding in both analyses.

The emission contribution of other inputs like chemical fertilizers and chemicals is quite low in both provinces. For example, the share of chemical fertilizers in total emissions is 0.26 kg CO_2_/ton in Konya, while it is 0.43 kg CO_2_/ton in Kırklareli. This indicates that the environmental impacts in cucumber production are largely linked to fossil fuel based energy consumption and that the use of chemical inputs results in a relatively lower environmental burden. However, a study by Özilgen and Sorgüven (2011) stated that the largest carbon emission contribution in vegetable oil production from sunflower and soybeans resulted from excessive fertilizer use^32^. Similar variations depending on crop type are observed in wheat production in Türkiye and Germany, where fertilization, irrigation, and diesel use were identified as the main emission sources, with Türkiye exhibiting higher values due to greater dependency on these inputs^36,37^. This comparison highlights that environmental impact profiles can vary significantly depending on the product type and agricultural practices used.

In conclusion, the findings indicate that the carbon emission profiles in greenhouse cucumber production in Kırklareli and Konya vary according to regional agricultural practices and climatic conditions. The higher emissions observed in Konya are primarily associated with the energy-intensive utilization of resources, specifically electricity for water extraction. Within the defined tillage-to-harvest assessment boundaries, these results suggest that strategies aimed at enhancing irrigation efficiency and exploring the integration of renewable energy particularly for operational electricity substitution could play a significant role in improving the environmental sustainability of regional greenhouse farming prototypes.

### CDP and RI analysis

The findings of this study are based on specific greenhouse conditions in the Kırklareli and Konya regions. Although these two regions represent different climatic zones, broader and long-term datasets are required to generalize the results on a global scale. Therefore, the presented sustainability improvements should be evaluated as a potential within the framework of the energy profiles of the studied regions.

CDP and RI are critical thermodynamic metrics that evaluate a production process’s sustainability by considering not only the quantity of energy inputs but also their quality and renewability level^40^. These metrics provide a powerful framework for comparing the environmental footprint and thermodynamic efficiency of production systems. In this study, the chemical exergy value was taken from the literature as 1.07 MJ/kg^39^.

As a result of the analysis, the CDP value for greenhouse cucumber production in Kırklareli province was calculated as 0.73, and the RI value as −0.36. In contrast, the CDP and RI values for Konya were determined to be 0.42 and − 1.36, respectively. Based on the calculated RI values, the greenhouse cucumber production process was found to be completely non-renewable for both Kırklareli and Konya provinces.

Kırklareli’s higher CDP value compared to Konya indicates that it uses input resources more efficiently. The main reason for this difference is that inputs like electricity and manure play a dominant role in cumulative energy and exergy consumption in Konya.

A study by Özilgen and Sorgüven (2011) stated that CDP values in high energy intensive processes like vegetable oil production ranged from 0.92 (soybean oil), 0.98 (olive oil), and 2.36 (sunflower oil)^32^. The inherently low chemical exergy of a product with high water content like cucumber (approximately 1.07 MJ/kg) generally limits CDP values. Taki and Yildizhan (2018) noted that the CDP for greenhouse cucumber production under Iranian climatic conditions was 0.23 but suggested that this value could increase to 0.47 with the use of renewable energy sources^39^. In this context, Kırklareli’s CDP value of 0.73 shows that it has reached a better level of thermodynamic efficiency than Konya, but still holds potential for improvement.

The calculated value of −0.36 for Kırklareli indicates that production is dependent on non-renewable resources and that the restoration work is higher than the useful work produced. However, Konya’s value of −1.36 reveals that this non-renewable dependency is much deeper. This difference is due to Konya’s agricultural production process consuming far more fossil fuels and electricity than Kırklareli.

A study on rice production by Nikkhah et al. (2021) stated that the most efficient rice variety had a positive RI value of 0.88, indicating that the process was partially renewable^41^. In contrast, Taki and Yildizhan (2018) calculated an RI value of −3.32 for greenhouse cucumber production in Iran, but stated that this value could increase to-1.09 with the use of renewable energy sources^39^. While these values show that greenhouse cucumber production in Türkiye is carried out with a more efficient agricultural process, the negative RI values in both provinces signal that serious steps need to be taken to increase the renewability of greenhouse cucumber production systems. Especially in Konya, high energy-intensive irrigation and fertilization practices are seen to negatively affect the RI value.

In conclusion, a comparative analysis of CDP and RI values shows that greenhouse cucumber production in Kırklareli is thermodynamically more efficient and less dependent on non-renewable resources environmentally than in Konya. However, the negative renewability indicators in both provinces clearly show that fundamental steps such as reducing fossil fuel consumption and integrating renewable energy sources must be taken to increase sustainability.

Based on the analyses and comparative evaluations, developing various strategies to increase the sustainability of the greenhouse cucumber production system will make the production process more efficient;

- ***Renewable Energy Integration***: Replacing natural gas used for greenhouse heating and diesel and electricity used in irrigation pumps with renewable energy sources will significantly reduce the environmental footprint. Türkiye’s high geothermal energy potential in some regions offers an alternative for heating. Solar energy, on the other hand, stands out as an effective solution for both electricity generation and water pumping. For example, a study examining date production recommends using solar energy instead of diesel fuel^40^. In models developed for rapeseed production, it has been shown that CExC and CCO_2_E values decrease significantly with the use of biofuels and solar energy^14^.
- ***Efficient Resource Management***: Reducing irrigation water, and consequently the energy spent on pumping, is of critical importance. Using efficient systems like drip irrigation instead of traditional methods such as surface irrigation will decrease water consumption and related energy costs. In fertilizer management, precision farming practices based on soil analysis can prevent excessive fertilizer use, thereby reducing both costs and environmental emissions.
- ***System Optimization***: Modeling techniques like the Multi-Objective Genetic Algorithm (MOGA) used in rapeseed production^14^ can also be applied to greenhouse cucumber production. Such an approach can help determine the most sustainable production conditions by striking a balance between maximum yield and minimum energy consumption and environmental impact. This way, an optimal point can be found to ensure that increases in input use are proportional to yield increases.

### Sensitivity analysis

**Fig. 5:**
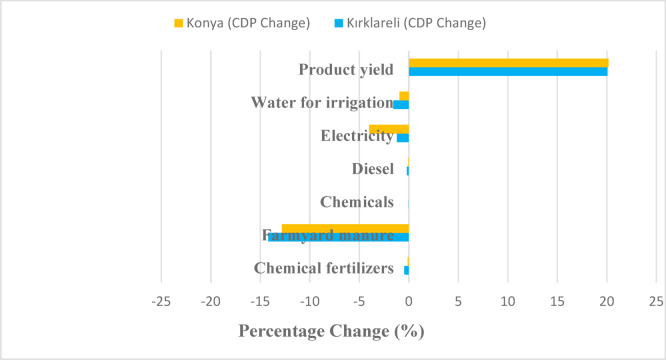
Sensitivity of the CDP to variation in input parameters and product yield for Kırklareli and Konya.

**Fig. 6:**
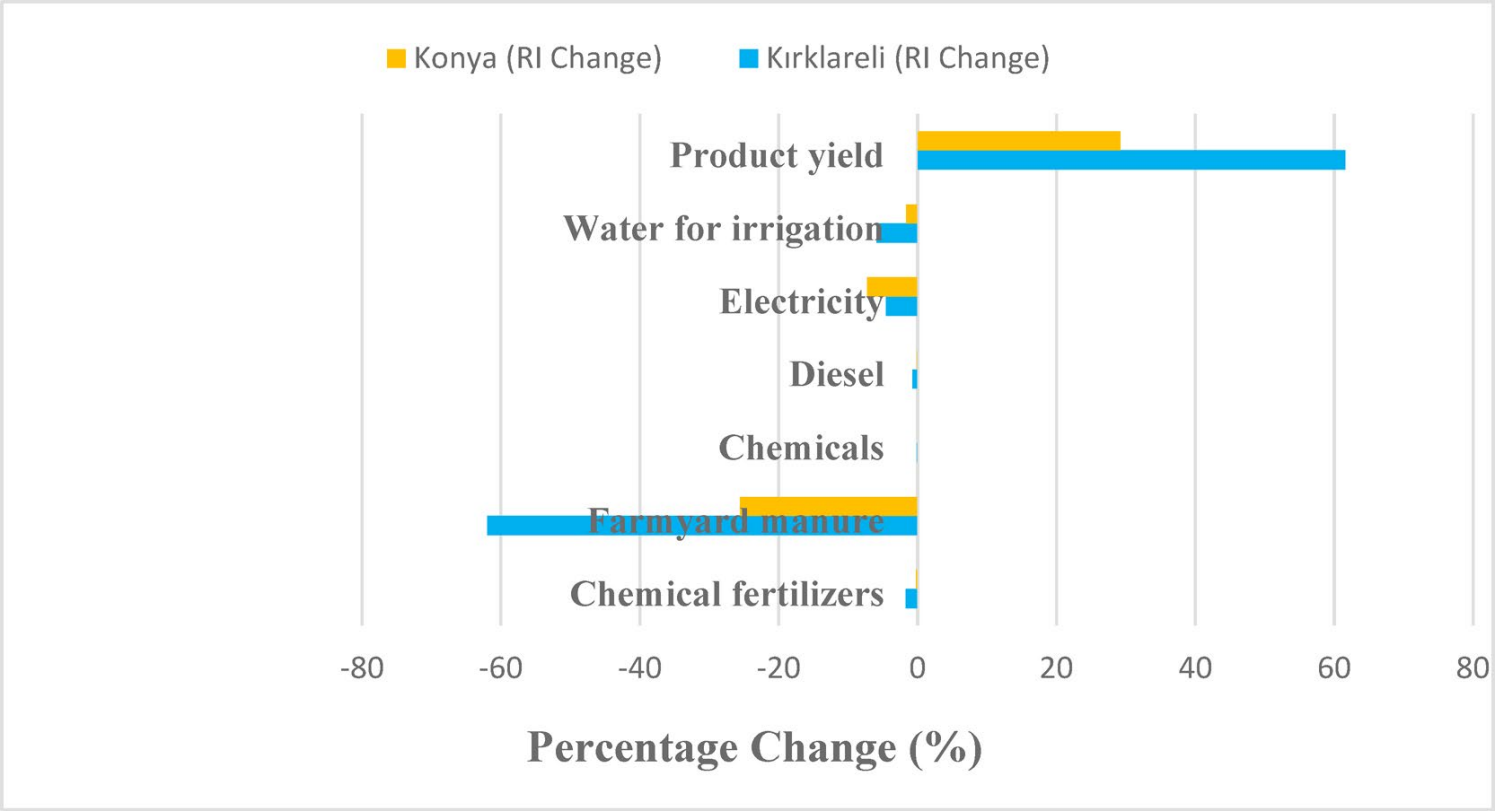
Sensitivity of the RI to variation in input parameters and product yield for Kırklareli and Konya.

In this study, a sensitivity analysis was performed to evaluate the stability and robustness of the sustainability indicators (CDP and RI) against a 20% variation in primary input parameters and product yield. The results, illustrated in Figs. 5 and 6, highlight which variables exert the most significant influence on the thermodynamic and environmental performance of the system in Kırklareli and Konya regions. The analysis reveals that product yield is the most critical parameter affecting system efficiency. A 20% increase in yield led to a linear 20.06% improvement in CDP for Kırklareli and a 20.17% improvement for Konya (Fig. 5). More remarkably, the same yield increase resulted in a 61.57% improvement in the RI for Kırklareli and 29.21% for Konya, emphasizing that operational productivity is the primary driver of renewability (Fig. 6).

Among the input categories, farmyard manure usage demonstrated the highest sensitivity, particularly impacting the CDP in both regions due to its significant exergy intensity. Conversely, the system showed minimal sensitivity to changes in chemical and diesel inputs, suggesting that the study’s conclusions remain robust despite potential minor uncertainties in these data coefficients. Although Konya’s RI exhibited a higher sensitivity to electricity variations (7.25%) compared to Kırklareli (4.54%), the overall regional performance hierarchy remained unchanged across all tested scenarios.

### The effect of PV panel use in greenhouses on CDP and RI indicators: a new scenario analysis

The PV integration scenario presented in this study focuses specifically on an operational electricity substitution model rather than a full life-cycle assessment. Consequently, the embodied energy of PV components, energy inputs for installation, detailed lifetime degradation rates, and the comprehensive carbon footprint of the PV infrastructure itself are considered beyond the scope of this work. This approach has been adopted to evaluate the thermodynamic impact of using renewable energy specifically for the energy-intensive stages of the greenhouse production cycle, from tillage to harvest.

Based on the regional energy demands identified in the previous sections, the feasibility of PV integration is evaluated particularly in light of Konya’s high electricity consumption for deep-well pumping.

**Fig. 7:**
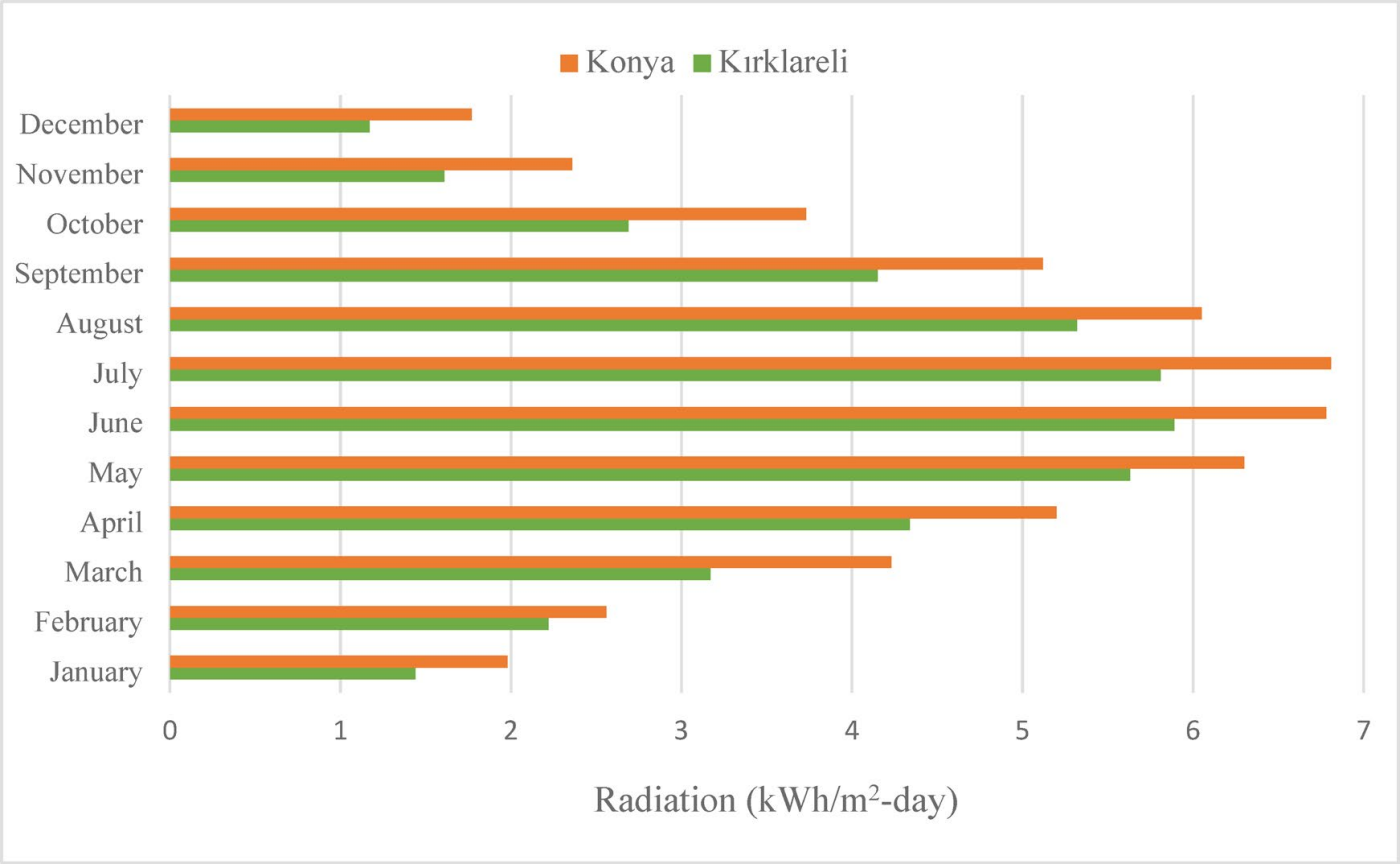
Solar radiation levels for Kırklareli and Konya provinces^42^.

**Fig. 8:**
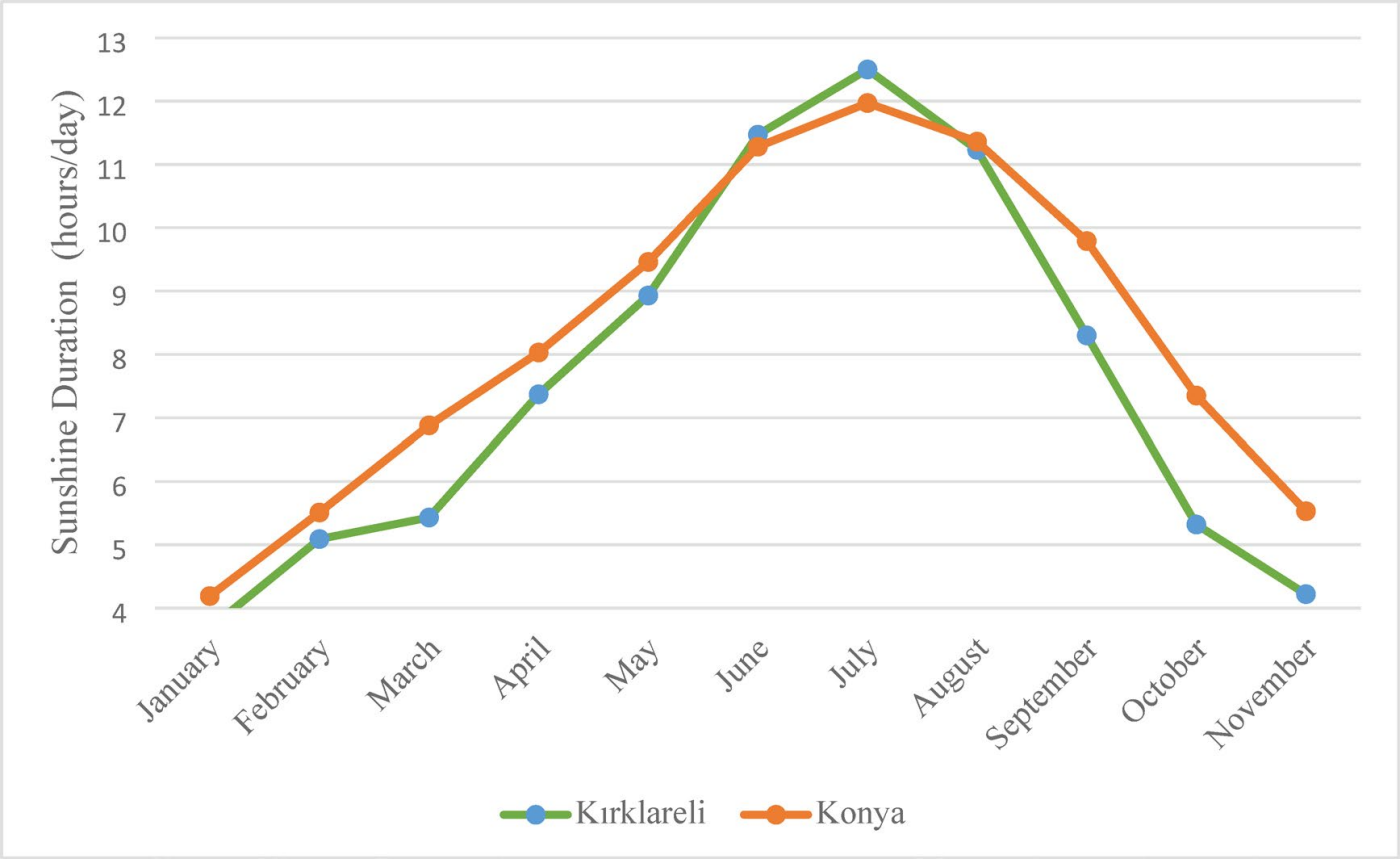
Sunshine duration values in Kırklareli and Konya provinces^42^.

Figures 7 and 8 show the monthly solar radiation and sunshine values for Kırklareli and Konya provinces. Konya’s annual average sunshine duration is 7.94 h and its global radiation value is 1608 kWh/m^2^-year. Kırklareli’s sunshine potential is a daily average of 7.2 h and a radiation value of 1321 kWh/m^2^-year^42^. These data show that the high cost of electricity consumption in Konya constitutes a significant burden, but at the same time, the abundant solar resource makes PV systems an extremely suitable and effective solution for the region. This is a key finding that reveals the direct relationship between climate, agricultural practice, energy consumption, and solar potential.

The new indicators obtained in the scenario where PV panels supply electricity show remarkable changes in terms of both system efficiency and renewability. In solar energy integration, the exergy consumption per 1 MJ of electricity production was taken from the literature as 0.19 MJ^30^. New values were calculated for the PV scenario according to this exergy consumption. Compared to the previous situation (Kırklareli: CDP = 0.73, RI = −0.36; Konya: CDP = 0.42, RI = −1.36), with PV integration, CDP increased to 0.77 and RI to −0.30 in Kırklareli; while in Konya, CDP rose to 0.51 and RI to −0.96. The PV panels meeting the electricity supply reduce the system’s non-renewable energy consumption and have a positive effect on CDP and RI. This transformation shows a potentially significant effect on renewability in Konya, while in Kırklareli, it could be complemented with broader measures such as input optimization and energy efficiency. These findings suggest the importance of exploring PV integration specifically for operational electricity substitution within greenhouse farming, while emphasizing the need for planning additional strategies tailored to local operational conditions.

## Conclusion

This study examined how the sustainability profiles of greenhouse cucumber production in Türkiye’s different climate regions vary depending on regional conditions and agricultural practices. The regional comparison revealed how production processes change according to differences in climate, irrigation infrastructure, and energy sources. In Konya, the energy required for water extraction and irrigation, as well as electricity use, significantly increases both CEnC and CExC. Kırklareli, with a lower energy intensive profile, performs relatively better.

For one ton of cucumber production in Kırklareli, CEnC was calculated as 260.16 MJ/ton, CExC as 1459.82 MJ/ton, and CCO_2_E as 31.13 kg/ton. In contrast, the values in Konya were calculated as 324.38 MJ/ton, 2521.12 MJ/ton, and 50.91 kg/ton, respectively. This difference is mainly due to the more intensive need for irrigation and electricity in Konya.

Exergy based sustainability indicators, with CDP at 0.73 and RI at −0.36 for Kırklareli and CDP at 0.42 and RI at −1.36 for Konya, reveal that production is highly dependent on non-renewable resources. The operational electricity substitution scenario via PV integration showed a potential for improvement, particularly in Konya, where CDP rose to 0.51 and RI to −0.96. In Kırklareli, CDP increased to 0.77 and RI to −0.30. Exergy based indicators (CDP, RI) showed how sustainable production processes are not just in terms of total consumption, but also in terms of resource quality and renewability.

The operational electricity substitution scenario through PV integration showed a potential to positively influence CDP and RI indicators, with more notable improvements observed in regional prototypes characterized by higher solar potential, such as the Konya case. In addition, adopting certain agricultural practices, including the transition to drip irrigation, enhancing pump efficiency, and optimizing fertilizer management, appears to offer a viable pathway for reducing energy and exergy consumption within the defined production boundaries. Consequently, the integration of energy and exergy-based assessments into regional greenhouse management (alongside efforts to mitigate carbon emissions and explore renewable energy applications) provides strategic insights that could contribute to the broader objectives of agricultural sustainability in Türkiye.

Future works should expand this boundary by incorporating the embodied energy of PV components, energy requirements for infrastructure installation, and long-term degradation effects. Conducting a comprehensive life-cycle exergy and carbon footprint analysis of the agrivoltaic systems themselves will provide a more holistic understanding of the net environmental benefits and long-term sustainability of integrated greenhouse operations.

## Data Availability

The data supporting the findings of this study can be accessed from corresponding author upon reasonable request.

## Abbreviation

CEnC: Cumulative energy consumption, MJ/ton
CExC: Cumulative exergy consumption, MJ/ton
CCO2E: Cumulative carbon dioxide emission, kg/ton
CDP: Cumulative degree of perfection
RI: Renewability Indicator
PV: Photovoltaic
h: Enthalpy, kJ/kmol
Q: Heat, kJ
b: Stream availability, kJ/kmol
m: Mass, kg
T: Temperature, K
W: Work, kJ

## Subscripts

0: Dead state
k: Index of heat sources
in: Inlet
out: Outlet
